# Energy Absorption Capacity in Natural Fiber Reinforcement Composites Structures

**DOI:** 10.3390/ma11030418

**Published:** 2018-03-13

**Authors:** Elías López-Alba, Sebastian Schmeer, Francisco Díaz

**Affiliations:** 1 Departamento de Ingeniería Mecánica y Minera, Campus las Lagunillas, Universidad de Jaén, 23071 Jaén, Spain; fdiaz@ujaen.es; 2 Institute for Composite Materials (IVW), Kaiserslautern University of Technology, 67663 Kaiserslautern, Germany; sebastian.schmeer@ivw.uni-kl.de

**Keywords:** crash absorption, structural material, impact behavior, natural fiber, specific energy absorption

## Abstract

The study of natural fiber reinforcement composite structures has focused the attention of the automobile industry due to the new regulation in relation to the recyclability and the reusability of the materials preserving and/or improving the mechanical characteristics. The influence of different parameters on the material behavior of natural fiber reinforced plastic structures has been investigated, showing the potential for transport application in energy absorbing structures. Two different woven fabrics (twill and hopsack) made of flax fibers as well as a non-woven mat made of a mixture of hemp and kenaf fibers were employed as reinforcing materials. These reinforcing textiles were impregnated with both HD-PE (high-density polyethylen) and PLA (polylactic acid) matrix, using a continuous compression molding press. The impregnated semi-finished laminates (so-called organic sheets) were thermoformed in a second step to half-tubes that were assembled through vibration-welding process to cylindric crash absorbers. The specimens were loaded by compression to determine the specific energy absorption capacity. Quasi-static test results were compared to dynamic test data obtained on a catapult arrangement. The differences on the specific energies absorption (SEA) as a function of different parameters, such as the wall thickness, the weave material type, the reinforced textiles, and the matrix used, depending on the velocity rate application were quantified. In the case of quasi-static analysis it is observed a 20% increment in the SEA value when wove Hopsack fabric reinforcement is employed. No velocity rate influence from the material was observed on the SEA evaluation at higher speeds used to perform the experiments. The influence of the weave configuration (Hopsack) seems to be more stable against buckling effects at low loading rates with 10% higher SEA values. An increase of SEA level of up to 72% for PLA matrix was observed when compared with HD-PE matrix.

## 1. Introduction

The automotive industry is focused on increasing energy saving, through different and/or more efficient on-drive systems, as well as the lightweight construction of vehicles. Nowadays, materials that are needed for structural components must present several properties to achieve technical requirements for the transport automotive industry. Composite materials have focused the attention of many car manufacturers since they offer a wide range of material properties values, and thus the requirements of the industry by combining two or more materials. An example of composites is fiber-plastic composites. They are mainly produced in the automotive industry by resin injection process and consist on a coherent matrix (thermoset or thermoplastic) and disperse fibers (carbon, glass, aramid or natural fibers). Furthermore, a significant change is taking place in the renewable resource. As it was defined by the European environmental directive 2005/64/EC [1], 85% of the car components must be recyclable and 95% reusable. Thus, there is an increasing interest in composites, such as flax fiber-reinforced polymers. Flax fibers present advantages, as a typical representative of plant-based natural fibers for polymer composite. This is because it can offer the best potential combinations of low cost, low density, comparable specific tensile properties to synthetic glass and worldwide availability. Therefore, they offer considerable potential for lightweight construction [2]. At the same time, natural fiber reinforced plastics are completely biodegradable if they are prepared with a biodegradable matrix, such as PLA (polylactic acid) [3,4].

Flax fiber is a natural fiber and therefore biological material, however it was observed that it has better mechanical properties compared with other non-biological material by external conditions and can thereby differ strongly in their mechanical behavior from each other [3,5]. Moreover, its mechanical behavior [6] can be substantially improved depending on the growing conditions in the field, as the fiber production process and the working conditions of the material. Mechanical properties of flax fiber reinforce composites were previously studied under quasi-static events [3,7,8] showing that the material properties are similar to glass fiber reinforced composites [9]. However, in reality, the structures are under different loading conditions, such as short-time dynamic loads that occurred during an impact, thus it is necessary to investigate the influence of the material stiffness and strength in case of high loading rates effect compared to static behavior. In addition, the energy absorption capacity of these materials it is an important issue to be study. The kinetic energy absorbed can modify the design of some component to avoid passenger’s injury or damage in the surrounding structures. In the last years, further steps were performed to study the energy absorption in natural fiber composites. Eshkoor et al. [10] showed the dependence of triggering mechanism configuration can cause significant differences in which the crashworthiness parameter and failure progress in natural silk epoxy composite tubes. Using the same material, Ataollahi et al. [11] explored the influence of the wall lengths on the compressive response using square tubes for energy absorption and failure response. Meredith et al. [12] explored the SEA influenced by fiber volume fraction and the variability resulted from the variation in fiber strength as results of the manufacturing process employed. They concluded that unwoven hemp exhibited an SEA value (54.3 J/g) that was comparable with carbon fiber (55.7 J/g). Yan et al. [13] investigated the effect of triggering and foam-filler and their effect on the crashworthiness characteristics of flax fabric reinforced epoxy circular composite tubes. In terms of SEA, it was concluded that the triggered and foam-filled tubes had these values larger that tube with triggering only. However these values of triggered and foam-filled tubes can be larger or smaller than the tubes with foam-filler lonely. In other study, Yan et al. [14,15] analyzed how foam filled tubes with more fabric plies exhibited better crashworthiness when compared to the empty tubes, showing that can be comparable to that of conventional aluminum tube and glass/carbon composites tube as energy absorbers. The aim of the following paper is to contribute to a better understand of the crash behavior of natural fiber-reinforced plastics for a better design of the components decreasing their development times and the cost. Only a few studies were found to be related to the mechanical performance of flax fiber reinforcement during impact. Rodriguez et al. [9] showed that flax fiber based composites exhibited a highest impact energy absorption when compared with the impact response of different natural fibers and glass fiber reinforced composite. Bledzki et al. [16] studied the load velocity impact response in thick flax/epoxy composite plates under the influence of the fiber content and void fraction, improving the absorbed energy related to an increment in the fiber content. Siengchin et al. [17,18] investigated the reduction of the peak force under impact test as a consequence of the nanoparticles presence on flax woven composites. Liang et al. [19] observed no significant differences in thin quasi-isotropic flax epoxy composite specimen under drop-weight impact test in a rate of 3.33 × 10^−5^ m/s and 2.98 m/s when considering that the impact response could be predicted by the quasi-static response. Bax et al. [20] investigated the impact behavior of PLA/flax and PLA/Cordenka composites suggesting that the last one presents better impact properties (72 kJ/m^2^). Nevertheless, further research was required to optimize the composite production parameter. Meredith et al. [21] investigated the Specific Energy Absortion (SEA) of Biotex MTM49 and Cordenka MTM49, demonstrating that Biotex flax combined with MTM 49 matches the SEA of T300 carbon fiber using the same resin system at 35 kJ/kg. However in this case Cordenka MTM49 did not demonstrate higher SEA with 23 kJ/kg, concluding the authors the potential of the natural fiber composite for its use in structural applications, but remarking the additional work required on fiber to matrix bonding in order to maximize their properties maintaining an environmental option. 

The aim of this paper is to investigate the impact behavior at different velocity impacts of three types of Biotex^®^ natural flax fiber and two different thermoplastic matrix HD-PE (high-density Polyethylene) and PLA (polilactic acid). Therefore, results of dynamic tests using a catapult device are compared to static reference test data. Different test configurations and parameters has been analyzed that to increase the specific energy absorption (SAE), improving the knowledge of these materials and their potential use in the transportation industry.

## 2. Materials and Specimens

In the present work, seven different materials that were manufactured using natural fiber reinforced composites have been investigated. Three different woven and non-woven technical textiles have been used as reinforcement; Twill (Flax 2 × 2, 420 g/m^2^) and Hopsack (Flax 4 × 4, 510 g/m^2^) from BIOTEX^®^ (Hamburg, Germany) and as non-woven mat (hemp/kenaf 1200 g/m^2^) from Dittrich&Soehne Vliesstoffe (Ramstein-Miesenbach, Germany). As matrix, PLA (polilactic acid) with a density of ρ_PLA_= 1.24 g/cm^3^ and HD-PE (High-Density Polyethylen) with a density of ρ_HD_-PE__ = 0.94 g/cm^3^ were used in foils. Table 1 and Table 2 list the different configurations and the nomenclature (V5–V11) and their properties used in this study. The fiber volume fraction of each material type was measured using the volumetric interaction method, and the results are shown. Basic material characteristics and mechanical properties of the materials used are indicated. In the row material composition, the number of dry fiber layers and the number of foil layers that were used for organic sheet production is given.

All of the materials usually differ in just one material parameter. V5 can be seen as a basic reference material for most of the configurations. V6 uses different weave configuration, V7_a different matrix type, and V10_a different wall thickness respect to V5. V11 is a non-uniform reinforced material. V8 differs to V7 and V9 to V6 in wall thickness. 

**Figure 1:**
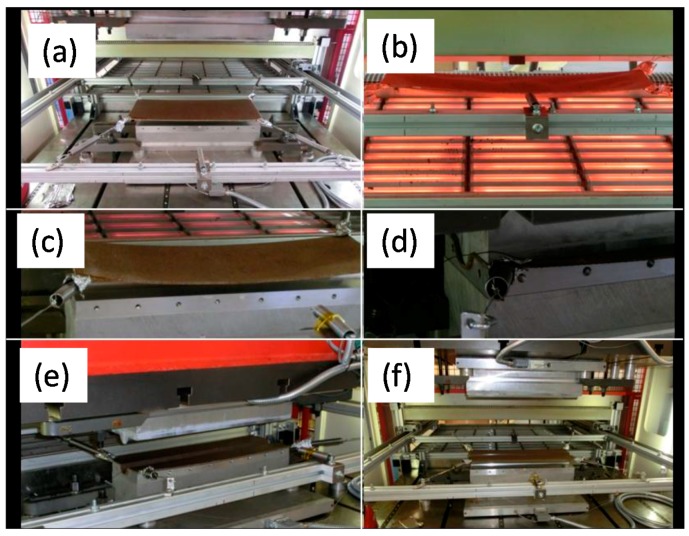
Forming process of the organic sheets. (**a**) deposit; (**b**) heating; (**c**) positioning; (**d**) forming; (**e**) cooling and (**f**) removing.

Organic sheets (V5–V10) using a continuous compression process were produced by impregnating dry fabric layers with two different polymers that are provided in foils. V11 was manufactured using static press. After that, specimen halves were produced by thermoforming of the organic sheets. This step is divided into heating, forming, cooling, and trimming. The process parameters were obtained from the bibliography [22]. Figure 1 shows the forming process by steps. 

The non-woven mat (V11) can not be alternately made with plastic film in the organic sheet manufacturing process, and therefore it has a deviating low welding time when compared to the other materials.

**Figure 2:**
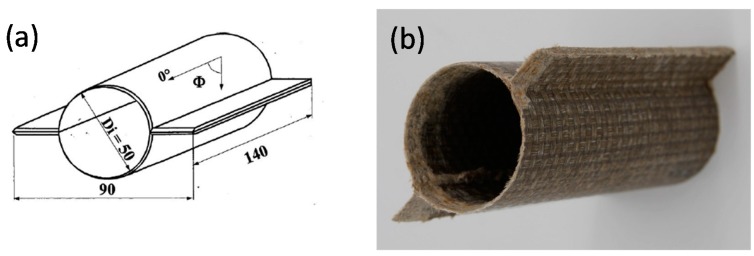
Specimen dimensions in mm for crash-tubes: (**a**) sketch of the specimen geometry; (**b**) specimen picture.

Finally, both halves of the specimen were assembled by a vibration welding system. The final steps for the production of crash test specimens are cut to size (length 140 mm, width 90 mm), which was carried out by end-milling cutter. Figure 2 illustrates the geometrical dimensions of the specimen crash-tubes. At one end a 45° trigger was generated.

Table 3 shows the mass of the specimens tested.

Once the specimens were prepared, experiments were planned to study the influence of the specific energy absorbed depending on the type of the fiber, the matrix the weaving, the wall thickness, and the absorption mechanism. 

## 3. Experimental Set-Up

To study the specific energy absorption behavior of natural fiber reinforced compositesm seven materials (V5–V11, Table 1) were tested at different test speeds (quasic-static and dynamic testing). Every test batch consists of three specimens in order to guarantee a minimum statistical coverage to absorb failed experiments. It is important to highlight that experiments at quasic-static speed allow for investigating different energy absorption modes in a more precise way than higher loading rates. However, the influence of the load and the strain rates were crucial for the experiments that were conducted. 

### 3.1. Quasi-Static Testing

**Figure 3:**
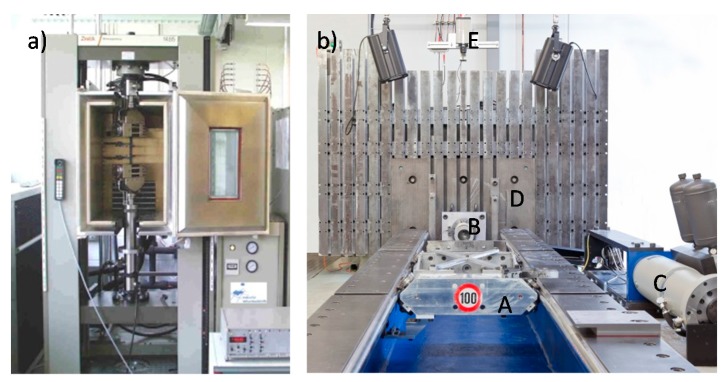
Universal testing machine (**a**) Quasi-static tests set-up. (**b**) Catapult crash facility used for dynamic tests.

Static tests for different materials were conducted on a Zwick universal testing machine (model Typ 1485, Zwick GmbH, Ulm, Germany) with a cross-head speed of 60 mm/min. as it is shown in Figure 3a. Specimens were vertically positioned on the grip compression plates. The cylinder’s displacement and thus the displacement experienced by the specimens were recorded to evaluate the specific energy absorbed. A CCD Redlake camera (model MotionPro HS3, nac Deutschland GmbH, Stuttgart, Germany) with 1280 × 1024 pixel sensor and a 50 mm focal lens (model Navitar TV Lens, Navitar, New York, NY, USA) was positioned to film the behavior of the structures during the tests. Quasi-static tests results are marked with the index “a” in next sections.

### 3.2. Dynamic Testing

A horizontal crash test facility was employed for dynamic testing at higher velocities. The crash test facility consists of a horizontal acceleration section, a crash rig with an impactor plate (A), a load cell (B), a hydraulic linear cylinder (C), a reaction mass (D), and a high-speed camera (E) (Figure 3b). The specimen was located upon the load cell. An accelerated mass was used to crash the specimens at a defined kinetic energy. Tests with an impact mass of 290 kg and an impact velocity area from 1.9 m/s to 3.3 m/s were performed. In a second batch of experiments, specimens were loaded with an impact mass of 61 kg and an impact velocity area from 4.2 to 9.1 m/s. Velocity areas were selected according to the material performance during quasi-static tests. The aim was to deform the specimens to 50–60% of the original length to perform a subsequent analysis of the deformation. A photocell measures the time between two light points evaluating the speed just before the impact. 

The impact plate of the carriage was marked with a target point, which was recorded with a CCD camera (Redlake model MotionPro HS3, nac Deutschland GmbH, Stuttgart, Germany), recording up to 64.000 fps using a 50 mm focal lens (model Navitar TV Lens, Navitar, New York, NY, USA). The material deformation was evaluated analyzing the position of the crash sledge. Results were labelled with the index “b” for an intermediate crash speed and index “c” for the highest crash speed employed.

### 3.3. Evaluation of Experiments

For each experiment, the force and the deformation are recorded and plotted. The area below that curve represents the total absorbed energy $E_{t}$ with $E_{t}$=$\int_{\Delta s}^{\;}F\left(s\right)ds$. To compare different geometries and materials the specific absorbed energy (SEA) is introduced with SEA = $E_{t}$/$m{\;}_{d}$. Where $m{\;}_{d}$ represents the “destroyed” mass. Since the cross section is constant through the height $m{\;}_{d}$ is calculated from the total mass $m{\;}_{t}$ (specimen’s weight) by using the relation between maximum deformation $m{\;}_{max}$ and specimen’s length l with $m{\;}_{d}\;$=$\;(m{\;}_{max}$/l)$\;m{\;}_{t}$. A very small amount of elastic stored energy is included in the energy absorption term, thus, this effect is negligible.

## 4. Results

A total of 63 experiments in batches of three specimens were performed to investigate and compare the potential of the chosen bio based materials for its use as energy absorbing structure. Therefore, the “characteristic curves” obtained from different batches of experiments were used. With the characteristic curves it is was intended to evaluate stable progressive energy absorption modes free of effects that are related to welding or material defects.

Two main failure modes can be distinguished in the experiments: catastrophic failure and progressive failure. Catastrophic failure modes are very inefficient for energy absorption since the material is only partly destroyed and the crushing load level drops down significantly after the catastrophic failure at the peak load. This failure occurs with unstable intralaminar or interlaminar crack growth through the material or global buckling because of the column instability. In any case a catastrophic failure should be inhibited and progressive failure should be aimed for. Progressive failure of continuous fiber reinforced plastics is a stable energy absorption mechanism that is based on four different failure modes that were investigated in detail in [23]. Transverse shearing (fragmentation), lamina bending (splaying mode), brittle fracture, and progressive folding. It should also be mentioned that friction between the material and the impactor also absorbs energy.

The materials that were employed in this work are V5–V11, as described in Table 1. Those differ usually in just one material parameter. These differences and the results rate are evaluated depending on the loading rate to quantify the SEA. 

**Figure 4:**
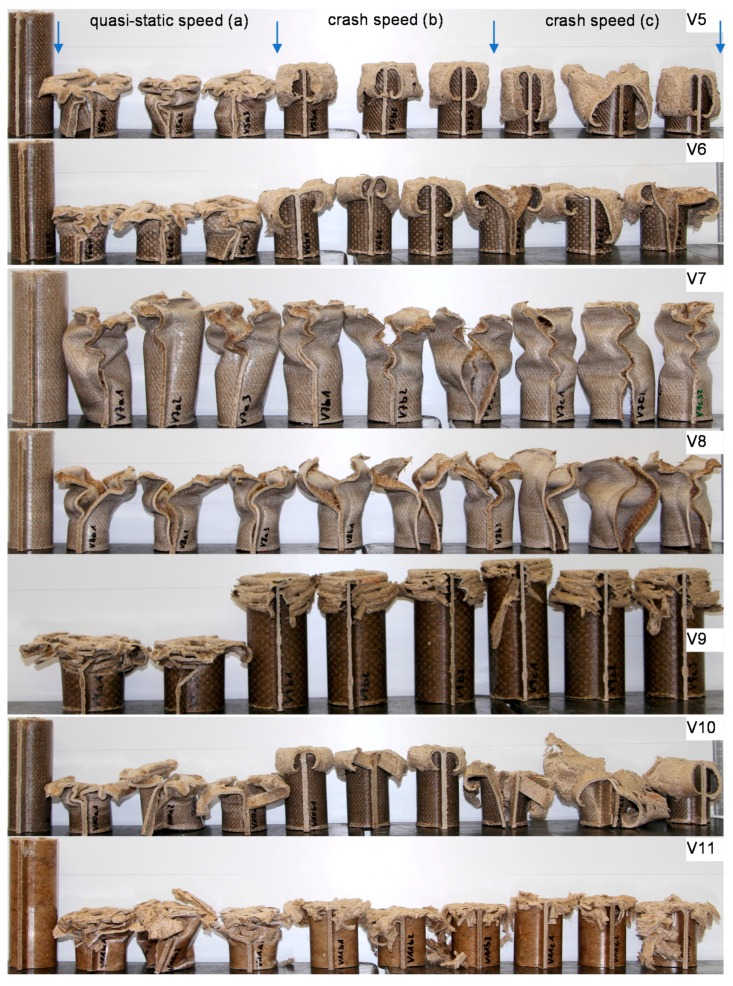
Deformed specimens of test series V5–V11. Left: undeformed specimen; middle: specimens tested at quasi-static speed; right: specimens tested at crash speed.

Pictures of the tested specimens are shown in Figure 4. The shapes of the deformed specimens in combination with the load deformation curves help to identify the energy absorption mode.

### 4.1. Quasi-Static Results

**Figure 5:**
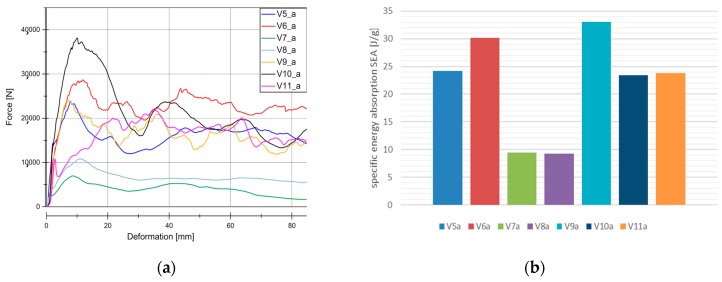
Results for quasic-static experiments (series “a”). (**a**) Load-Deformation curve for all the material under quasi-static conditions. (**b**) SEA for all of the material under quasi-static conditions.

Results from quasi-static tests (labelled with index “a”) are shown in Figure 5. The responses of the materials are displayed in force-deformation curves, Figure 5a. A pronounced F_peak_ at the beginning of crushing is reached in all of the materials, except for V11. This is an indicator of the predominant failure by progressive folding failure. This can be also confirmed by the shape of the tested specimens (Figure 4). Materials with the HD-PE matrix (V7 and V8) show remarkable lower profile stiffness and a significant lower crushing load level. The stiffness of HD-PE does not seem to be able to make stable natural fibers against local buckling. Thus, the fibers potential cannot be exploit with HD-PE matrix. Increasing the wall thickness leads to higher crushing levels (V5–V10 and V7–V8) and decreasing the wall thickness results in a lower crushing load level (V6–V9). Evaluating the Specific Energy Absorption (SEA) referred to the quasi-static experiments in Figure 5b, V9 reaches the highest SEA (32 J/g). The failure mechanisms in V9 are based on a mixture of progressive folding and lamina bending.

**Figure 6:**
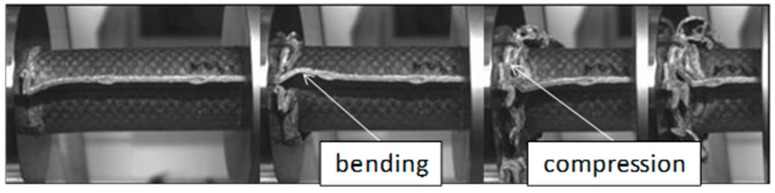
Deformation of V9 at quasi-static test.

Specimens with the Hopsack weave configuration (V6, SEA 29 J/g and V9, SEA 32 J/g) seems to be more stable against progressive folding and therefore, they ends up in a little higher SEA related to Twill weave specimens (V5, SEA 23 J/g), Figure 6. Thus, the wall thickness has no significant influence on the energy absorption potential. The force increase described in Figure 5 is proportional to the mass increase and very similar SEA values are evaluated at different wall thicknesses (V5, SEA 23 J/g) ‒> V10, SEA 23 J/g), (V6, SEA 29 J/g ‒>V9, SEA 32 J/g), and (V7, SEA 9 J/g ‒> V8, SEA 9 J/g). As indicated in the force-deformation diagrams, PLA matrix based materials reach 2.5 times higher SEA values compared to HD-PE matrix based materials.

### 4.2. Dynamic Results

**Figure 7:**
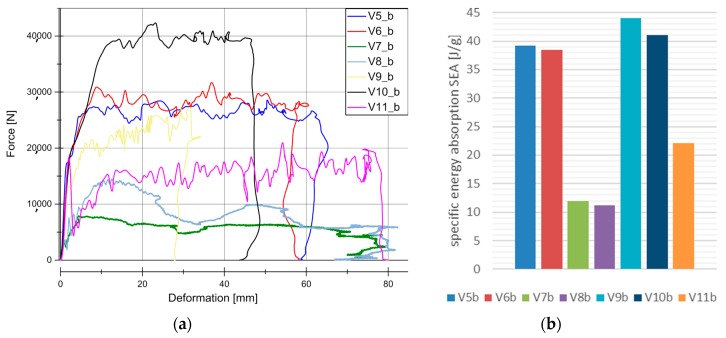
Results for dynamic experiments (series “b”). (**a**) Load-Deformation curve for all the materials under dynamic conditions; (**b**) SEA for all the material under dynamic conditions.

**Figure 8:**
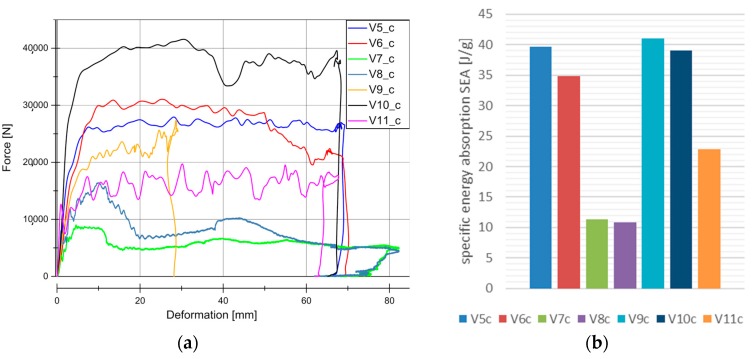
Results for dynamic experiments (series “c”). (**a**) Load-Deformation curve for all the material under dynamic conditions. (**b**) Specific Energy Absortion (SEA) for all of the material under dynamic conditions.

Figure 7 and Figure 8 illustrate the force vs deformation results for the crash tests performed (series “b”: 1.9–3.3 m/s and series “c”: 4.2–9.1 m/s). In general very similar principle effects at both investigated velocity regions can be observed. At these higher loading rates, a force peak occurs only for V7 and V8. The very homogeneous force level is an advantage for an application in crash absorption because severe oscillations would have to be compensated by surrounding components. 

Figure 7b and Figure 8b show the evaluation of SEA values of the investigated specimens at series b and c. It is remarkable, that V5 (SEA 39 J/g) reached comparable SEA values to V6 (SEA 38 J/g) at this loading rate in contrast to quasi-static velocity. In all of the cases for PLA based matrix SEA values increased with a higher loading rate. This is directly connected to the described change from progressive folding dominated absorption mode to laminate bending dominated mode. The reason for that change in the absorption mode is the increased stability of the part against local buckling caused by inertia effects. 

**Figure 9:**
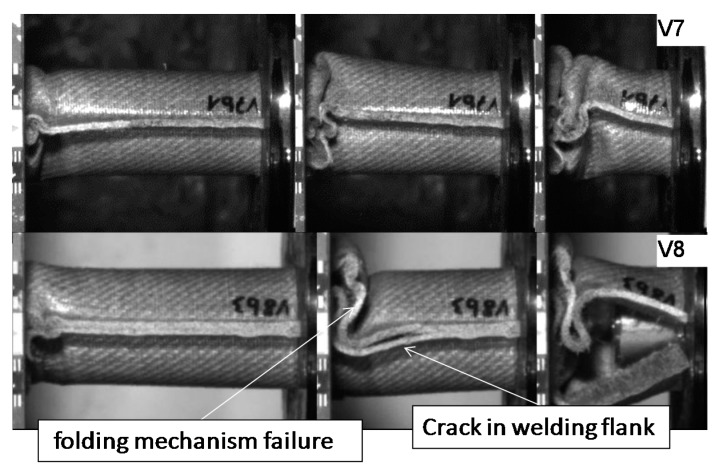
Failure mechanism for V7 and V8 in crash tests series b.

The wall thickness increase had no influence on SEA values (V5, SEA 39 J/g ‒> V10, SEA 41 J/g and V7, SEA 12 J/g ‒> V8, SEA 11 J/g). Just a decrease in the wall thickness from V6 (SEA 38 J/g) to V9 (SEA 44 J/g) leads to a detectable increase in SEA. The lower wall thickness of 1.75 mm provides an optimum relation between buckling stability and low mass. In parallel to quasi-static results and inferred from the force-deformation curves a change of matrix from PLA to HD-PE result in very low and unattractive SEA values. Similar results were obtained for higher velocities rates in series c experiments. It has to be considered that in some series, a welding failure occurred in all of the investigated specimens. This is a consequence of an energy absorption mechanism primarily based on progressive folding, as it can be also confirmed in high speed pictures in Figure 9. The enlarged wall thickness in V8 specimens caused a higher resistance against folding leading to higher forces and to fail at welding area for all specimens. This can be also observed looking at the force deformation curves at the two force drops at deformation values that were close to 23 mm and 55 mm from series. This effect can be also observed series c (Figure 7a and Figure 8a).

**Figure 10:**
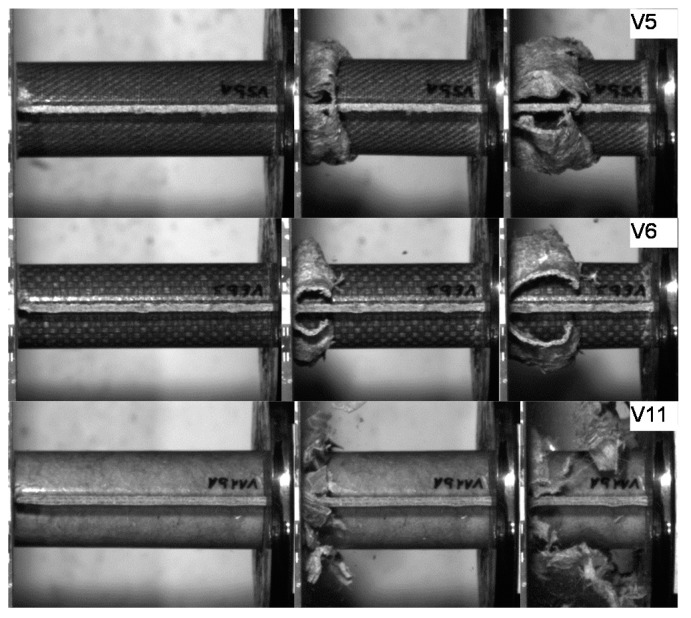
Failure mechanism of V5, V6 and V11 in crash tests series b.

In the PLA based materials with continuous fiber reinforcement V5, V6, V9, and V10, the main failure mechanism was laminate bending. As an example, crash sequences of V5, V6, and V11 from series b are shown in Figure 10. In series c, a higher percentage of welding failure occurred probably because of the higher impact energy and the following wave propagating through the specimen. This can be seen in V6_c by a force decrease due to the welding failure at a deformation of 52 mm (Figure 7a). 

The undirected fiber reinforcement of V11 cannot cohere the material while crushing. Therefore, laminate bending absorption mode cannot be established, and the material fails under brittle fracture and fragmentation, as it is shown in Figure 10. This leads to a significant force drop after the first crushing failure (visible in series b). In series c, the force level stabilizes at the first force peak. 

**Figure 11:**
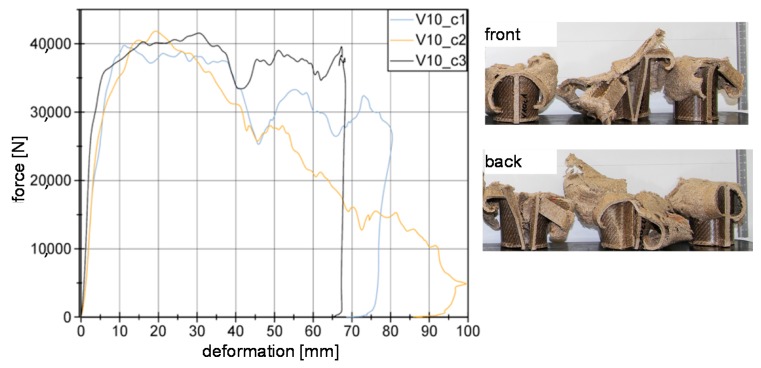
Series c of V10 material in force-deformation curves as an example.

Figure 11 shows the force-deformation curves for series c of V10 material. As it is shown, a crushing load level of around 40 kN. However, for specimen V10_c2 it is observed a failure in the welding area (front) starting at around 25 mm, resulting in a constant decrease in the load level. Specimen V10_c1 remains stable up to 40 mm. There, a failure in the welding area at the back appears. 

## 5. Discussion

In this section, a discussion of results that are related to the different material tested is performed. This discussion has been based on different parameters, such as: the loading rate influence, the wall thickness, the weave configuration, the material textile reinforcement, and the matrix type.

### 5.1. Loading Rate Influence to Material Behaviour

The loading rate influence is significantly based on the energy absorption mode. For the case of quasi-static tests, a progressive folding absorption dominates all of the specimens except V11. Laminate bending mechanism reach more energy absorption effectiveness compared to progressive folding failure mechanism, this explains the significant differences between force-deformation curves for series a and series b/c in V5, V6, V9, and V10. No velocity rate influence from the material was observed on the SEA evaluation at the speeds used to perform the experiments b and c. Here, the dominating energy absorption mode is laminate bending. However, for V7 and V8 no influence in SEA is presented and no change in the energy absorption mode (progressive folding) occurs. For V11 the energy absorption mode is brittle failure and fragmentation for all the loading rates and therefore SEA values remain constant.

### 5.2. Wall Thickness Influence to Material Behavior

**Figure 12:**
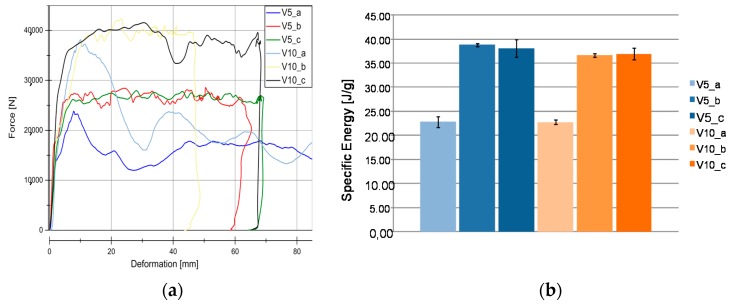
Results for V5 and V10 materials. (**a**) Load-Deformation curve of V5 and V10 material under different velocities rates in crash tests conditions. (**b**) SEA evaluation in V5 and V10 series (Twill/PLA wall thickness evaluation).

The influence of the velocity rates and the wall thickness is analyzed based on materials V5 and V10. Both are manufactured using reinforcing woven fabrics (twill made of flax fibers (Biotex^®^, Hamburg, Germany) and PLA matrix (see Table 1). Figure 12a illustrates the responses for the different test series in both of the materials. Specimens differ only in their wall thickness (V5 2.4 mm and V10 3.5 mm). Figure 12a shows the characteristic rectangular curves for V5 material at different strain rates b and c. However, a F_peak_ is only available with quasi-static experiments (series a). The reason is due to the fact that folding failure mechanism occurred. The V5 material shows a significant force increasing in series b and series c when compared with series a when the deformation increases. Similar F_peak_ forces were obtained by all of the series however for crash tests, the maximum deformation achieved was larger when compared with the quasi-static tests. Figure 12b shows a comparison of SEA values. It can be seen that no velocities rates for V5_b and c series were experienced. In contrast to this, in V10_a decreasing force at 35 mm deformation occurred for c series. As results, similar SEA values as compared with series b were obtained, as it is detected in Figure 12b. This effect can be explained due to the existence of cracks in the welding flanks. Therefore, at measuring range (maximum deformation) by a higher kinetic energy impact elevate the material error probability. In both cases it is assumed that the lower SEA occurred in “series a” under quasi-static tests. However, SEA in V10_a was slightly lower than V5. 

**Figure 13:**
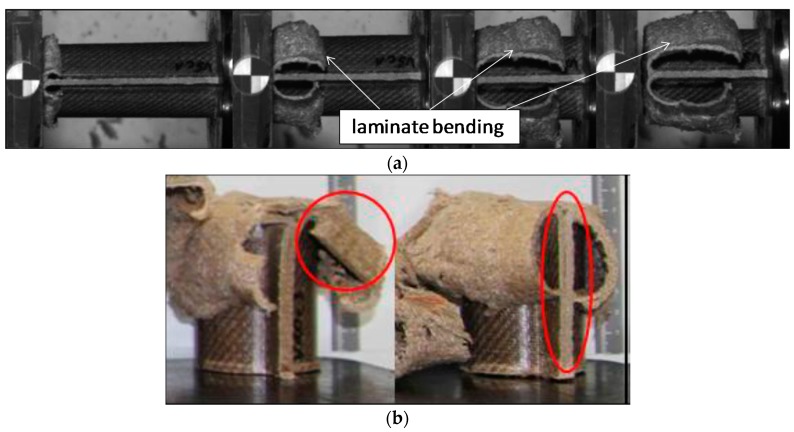
Results of failure mechanisms for V5 and V10 materials. (**a**) Laminate bending mechanism for V5_c during a crash test. (**b**) Rear and front part of V10_c specimen crashed.

As it was previously mentioned, in V5 material, it was observed a significant force increase in series b and series c when compared with series a when the deformation increases. This can be explained by the fact that V5 specimens experience different failure mechanisms due to the velocity rate effect. Laminate bending mechanism failure is presents in b and c as illustrated in Figure 13a based in the V5_c results. Laminates are experiencing a laminate bending, and therefore an almost negligible elastic deformation occurs. The same mechanisms failures were experienced for V10 specimens. Figure 13b illustrates the back sample sections (red circle) of V10_c, in this case, the sample does not absorb energy and the SEA value decreases due to the buckling failure produced. Cracks propagation were highlighted at the welded zone (ellipse).

As a conclusion from the force-deformation curves for V5_b, c and V10_b, c it can be deducted that a slightly SEA decrease is obtained as a function of the wall thickness from 2.4 mm (V5) to 3.5 mm (V10). Therefore, the reduction in D/t ratio results in an increase in the specific energy. This increase is due to a reduction in the interlaminar cracking in the crush region of the tube. Similar results were observed for other composite materials, such as carbon/epoxy and Kevlar/epoxy [24,25]. Other studies [26] in carbon fiber/PEEK tubes concluded that when t is in the range between 2 mm and 3 mm, all types of tubes display their highest specific energies. With an increasing t up to this critical range, the SEA value decreased. Similar results are extracted for V5 and V10 behavior. However, it is important to remark that some failures occurred during the crashing due to welding cracks in the flank for V10, thus furthers studies would be needed to improve the welding process.

### 5.3. Weave Configuration Influence to Material Behavior

**Figure 14:**
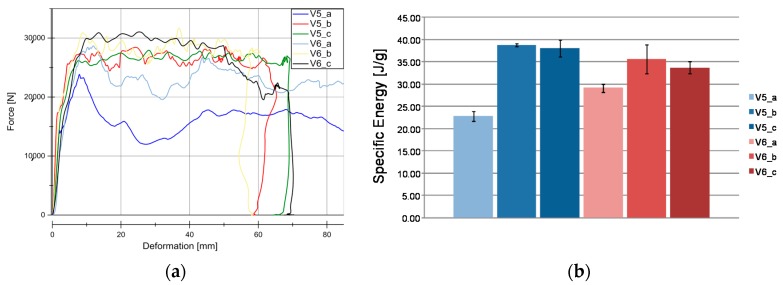
Results for V5 and V6 materials. (**a**) Load-Deformation curve for V5 and V6 materials under different velocities rates under crash tests conditions. (**b**) SEA evaluation in V5 and V6 series (Twill/PLA and Hopsack/PLA weave type evaluation).

The materials V5 (flax twill/PLA) and V6 (flax Hopsack/PLA) differ in their reinforcement structure. The influence of the weave configuration is examined in the SEA evaluation. Results for material V5 have been already explained and employed in the following as reference values. Hopsack weave configuration seems to be a little bit more stable against buckling effects at low loading rates (Figure 14a). This results in 10% higher SEA values for the progressive folding dominated energy absorption. Therefore, V6 material presents a lower velocity rate dependency than material V5. However, more important is the behavior in crash loading. Here, no significant difference between V5 (SEA in b, 39 J/g and in c, 40 J/g) and V6 (SEA in b, 38 J/g and in c, 35 J/g) can be observed. As it was mentioned before all specimens in V6_c tests were affected by welding flank failure, reducing the SEA value effect, as observed in Figure 14b.

**Figure 15:**
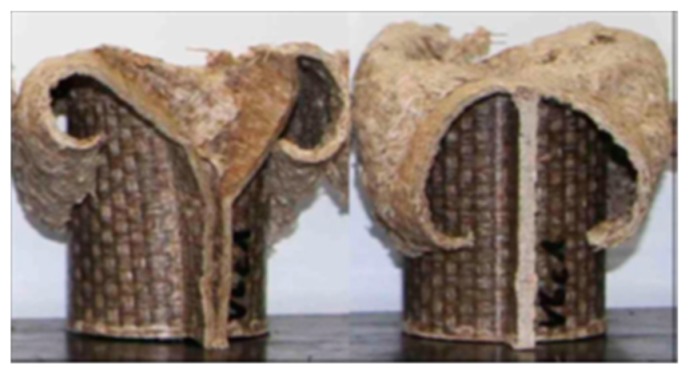
Rear and front part of V6_c specimen after crash test.

The failure that is illustrated in Figure 15 affects the SEA evaluation. Without welding failure, a similar velocity rate would show a laminate bending failure for series b and series c as in V5. V6 curves for series b and c show a similar behavior in the range until 50 mm deformation. Due to the presence of welding cracks in the V6 material, results shows some scatter. However it is saw that there is not a significant influence in the SEA evaluation for the weave configuration in Twill and Hopsack reinforcement fabrics. In the case of quasi-static analysis a 20% increment in the SEA value is observed when wove Hopsack fabric reinforcement is employed.

### 5.4. Reinforcement Textile Material Influence in SEA Results

**Figure 16:**
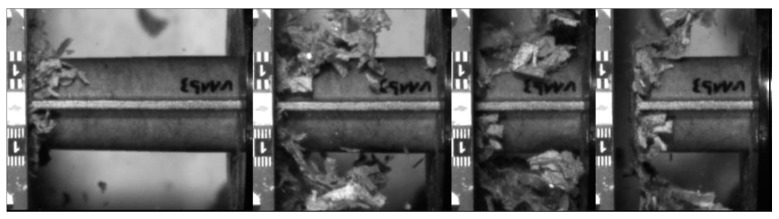
Transverse shear fracture failure mechanism for V11_b and c series experienced during the crash test.

Further examination of the reinforcing structure using the same matrix (PLA) has been conducted and a comparison between the V5 material (woven twill fabric) with the V11 material (non-woven Vlies) has been conducted. As it is observed in Figure 16, the V11 material was tested at the velocities rates b and c, and it was fragmented during the experiments. A brittle fracture was produced as a result of a mixture of transverse shear (dominant) and a laminate bending mechanism.

**Figure 17:**
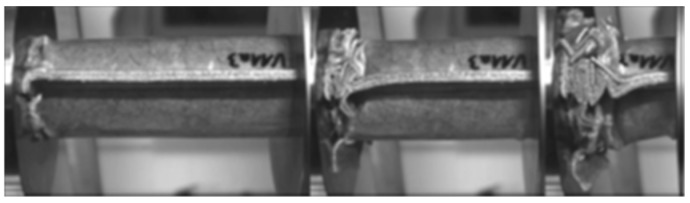
Failure mechanisms for V11 material in a quasi-static test.

In V11 material, the failure mechanism of series a, b, and c differ only slightly. As it was indicated by other authors, materials with brittle fracture sometimes have a low strain rate dependency [23,24]. During quasi-static test (series a), it was observed a mixture of brittle fracture and progressive folding. Figure 17 illustrates how the progressive folding failure mechanism was dominant at early stages of the crash experiment. After that, brittle fracture and folding failure mechanisms occurred simultaneously. As a consequence a higher SEA and force-deformation relation was obtained for V11_a when compared with series b and c. The reason is due to a progressive folding failure mechanism immediately after the first stage shown in Figure 17. During the test, brittle fracture appeared, however no fragmentation was observed, which explains the higher SEA results for V11_a series.

**Figure 18:**
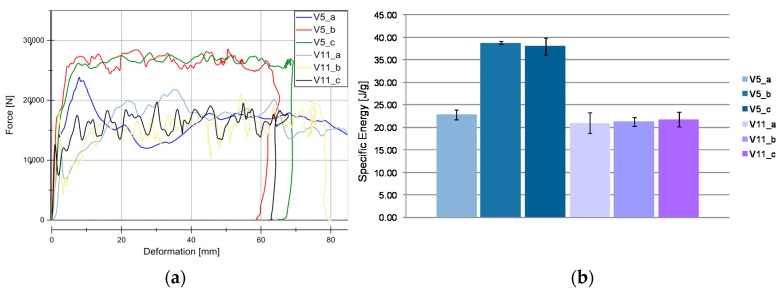
Results for V5 and V11 materials. (**a**) Load-Deformation curve for V5 and V11 materials under different velocity rates under crash tests conditions. (**b**) SEA evaluation for V5 and V11 series (Twill/PLA and Vlies/PLA reinforce influence evaluation).

Figure 18a shows that force-deformation curves for V5 and V11 materials. It is observed a higher force in V5 than in V11. For b and c series both materials show a fluctuation around the central force. When comparing the similarity between force functions for V5_a and V11_a, b, and c it can be deducted that the failure mechanism by brittle fracture and progressive folding for V11_a, b, and c is more efficient in terms of energy absorption than progressive folding appeared in V5_a series. However, a high velocity rate dependency between both materials exists. As a result, twill fabric samples body (V5) shows a higher energy absorption capability than the non-woven samples V11 (Figure 18b).

### 5.5. Matrix Type Influence in Material Behavior

**Figure 19:**
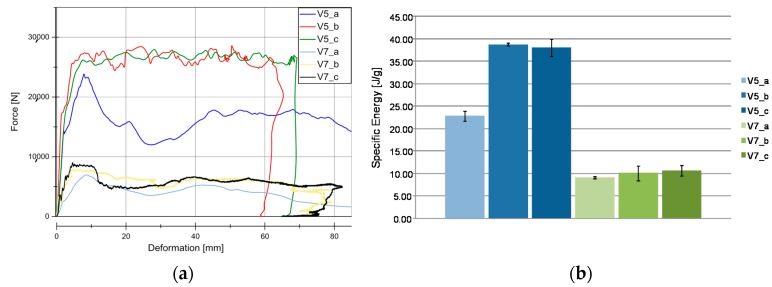
Results for V5 and V7 materials. (**a**) Load-Deformation curve for V5 and V7 materials under different velocity rates under crash tests conditions. (**b**) SEA evaluation in V5 and V7 (Twill/PLA and Twill/HD-PE matrix influence evaluation).

Once the influences by different parameters was analyzed, such as the wall thicknesses, the type of weaving and reinforcing structures, the influence of the matrix on the force-deformation curve, and SEA, the matrix influence is an interesting parameter to study. The V7 material (HD-PE) differs mainly in the matrix that is used in the manufacturing process, as compared with V5 (PLA). In Figure 19a, it can be seen that there is no velocity rate dependency in V7 series b and c. Despite almost the same structure of woven fabrics (twill) made of flax fibers (Biotex^®^) was used, the differences in forces levels are significant. V7 material series b and c only achieved a small fraction of V5 series b and c. This is also highlighted in Figure 19b, showing a comparation of SEA between both materials. It can be seen that V7 (series b and c) represent only 25% of V5. A generally accepted explanation is that the mechanical properties of the matrix (HD-PE and PLA), and their bond properties with the reinforcement flax-fiber structure. The V7 matrix has an insufficient rigidity, and thus the fibers cannot be sufficiently supported, reducing the stability and the SEA. Similar behavior was observed for V8 material.

**Figure 20:**
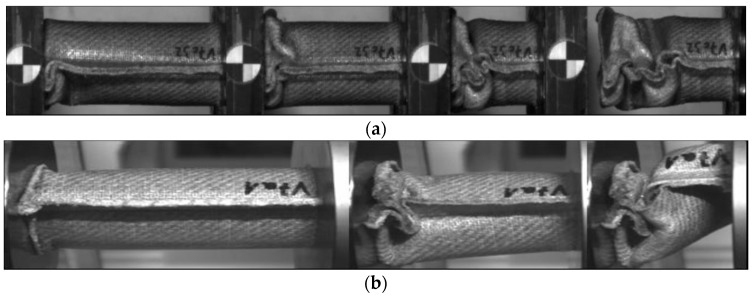
Failures mechanism for V7 in: (**a**) b and c series. (**b**) Quasi-static tests a series.

For V7 material, the results that were obtained from the quasi-static test (series a) shows that there is a smaller difference to series b and c in comparison with other materials, such as V5, V6, and V10. Similar behavior was observed for V11 material. This effect is explained by the fact that the failure mechanism (progressive folding) is the same in all the experimental tests in series a, b and c. Figure 20. It is remarkable that higher elastic energy is recovered when laminate bending (V5) or brittle facture (V11) failure mechanisms are involved, as it is shown in Figure 19a.

## 6. Conclusions

The aim of this study was to investigate the potential of natural fiber-reinforced plastics for energy absorption applications. For the analysis, two different woven fabrics (twill and hopsack) that were made of flax fibers as well as a non-woven fabrics made of a mixture of hemp and kenaf fibers were used as reinforcing materials. Two different matrix HD-PE (high-density Polyethylen) and PLA (polilactic acid) were used. Different configurations of these materials (V5–V11) were tested in order to investigate the influence of the wall thickness, the matrix type, and the weave configuration. Those experiments were conducted under quasi-static conditions (series a) and two additional crash speeds (series b and c) evaluating the influence of the loading rate during the experiments. All of the test series consisted of three specimens, with a total of 63 tests were run, evaluated and analyzed. 

Failure mechanisms due to crash loadings (series b) were mainly based on laminate bending (V5, V6, V9, and V10), progressive folding (V7 and V8), and brittle fracture (V11). The most effective energy absorption was reached by laminate bending dominated failure states. Under quasi-static loading (series a) failure is mainly dominated by progressive folding mechanisms. However, the energy absorption level is lower than for the case of crash loading. This different behavior is mainly influenced by the matrix.

The wall thickness influence was investigated by Twill fabric with PLA matrix (V5 and V10), Hopsack fabric with PLA matrix (V6 and V9) and Twill fabric with HD-PE matrix (V7 and V8). No significant influence of the wall thickness to SEA was observed for the investigated thicknesses between 2.2 mm and 3.5 mm. Just for V6 and the lowest wall thickness of 1.75 mm, an increase of SEA of 15% was observed related to the reference wall thickness of 2.7 mm. In all the cases of high SEA values, the laminate bending mechanism was reached. This seems to be an important requirement for stable energy absorption in these materials. 

The influence of the weave configuration was investigated for Twill fabric (V5), Hopsack fabric (V6), and non-woven reinforcement (V11). SEA values for V5 and V6 were within the same level, while V11 only reached 50% of fabric values. The energy absorption mechanism was brittle fracture and fragmentation for all of the investigated loading rates. However, it must be considered, that the fiber volume fraction in the non-woven material was only 28% compared to the fabric material with 50%/54%. 

The matrix influence on SEA was evaluated by the comparison of twill fabric with PLA matrix (V5) and twill fabric with HD-PE matrix (V7). Again, the matrix influence turns out to be relevant for SEA: The SEA for V7 and V8 reached the values of 11 J/g and 12 J/g, only a fraction of the material for the case of V5 and V6 with 39 J/g and 38 J/g, respectively. The lack of matrix stiffness and strength and thereby a loss in the support function of the matrix contributes to this behavior. 

The energy absorption behavior of natural fiber reinforced plastics is influenced by different material parameters as explained in this paper. It can be observed that continuous natural fiber reinforced materials based on PLA matrix reach SEA values over 40 J/g. The characteristic of the resulting curves. Since this value exceeds the SEA of steel and aluminum alloys, natural fiber reinforced PLA materials can be potentially considered as an alternative for energy absorption applications. 

## Figures and Tables

**Table 1:** Material characteristic and mechanical properties of the materials that were tested.

	Material Composition	Thickness (m)	V_f_ (–)	Area Density (g/mm^2^)	Modulus of Elasticity (N/mm^2^)	Ultimate Tensile Strength (N/mm^2^)	Ultimate Elongation (%)
V5	4 Twill + 5 PLA	2.4	0.5	3027.2	8711.4 +/− 278.8	73.8 +/− 2.2	1.66 +/− 0.13
V6	4 Hopsack + 5 PLA	2.7	0.54	3481.0	10289.0 +/− 206.8	88.5 +/− 2.5	1.63 +/− 0.10
V7	4 Twill + 5 HD-PE	2.2	0.55	2382.3	4348.8 +/− 58.9	58.9 +/− 1.3	3.06 +/− 0.25
V8	6 Twill + 7 HD-PE	3.15	0.57	3457.8	4554.1 +/− 350.9	61.7 +/− 0.9	3.12 +/− 0.34
V9	2 comingled Hopsack/PLA	1.75	-	2248.8	7329.5 +/− 214.4	37.2 +/− 3.9	0.83 +/− 0.19
V10	6 Twill + 7 PLA	3.5	0.51	4379.8	9278.8 +/− 298.7	83.4 +/− 2.3	1.59 +/− 0.22
V11	1 Vlies + 7 PLA	2.95	0.28	3333.9	-	-	-
PLA	-	-	0	309.6	3223.0 +/− 136.2	49.3 +/− 2.1	1.8 +/− 0.03
HDPE	-	-	0	188.3	943.5 +/− 15.5	21.1 +/− 0.3	10.13 +/− 0.29

**Table 2:** Material characteristic and mechanical properties of the matrix materials and typical plastics for comparison (CELC: Flax and Hemp fibres: a natural solution for the composite industry. JEC-Composite, 2012).

Synthetic Material	Density (g/cm^3^)	E-Modulus (GPa)	Tensile Strength (MPa)	Breakage Strain (%)	Tg (°C)	Ts (°C)	Typ
HD-PE	0.95–0.97	0.55–1.1	20–37	10–1200	−3	117	TP
PP	0.9–0.91	1.2–1.7	30–70	10–600	−20	−28	TP
PVC	1.3–1.6	2.4–4.1	40–60	40–80	77	157	TP
Polyester	1.1–1.4	1.3–4.5	45–85	1–5	67	-	TS
Epoxid	1.2–1.4	2.1–4.5	40–85	2–7	107	-	TS
PLA	1.21	3.3	30	2.5	37–67	127–177	TP Bio
PHB	1.18	3.5	40	5–8	2	137–177	TP Bio

**Table 3:** Specimen’s weight.

Material	Mean Mass (g)	St dev.
V5	91.3	1.6
V6	104.5	0.9
V7	72.1	0.8
V8	107.0	0.9
V9	68.4	0.6
V10	131.7	1.6
V11	96.2	1.5
